# Influence of Preparation and Exposure Periods of Eluates from Ocular Prosthesis Acrylic Resin in Human Conjunctival Cell Line

**DOI:** 10.29252/.23.1.78

**Published:** 2019-01

**Authors:** Emily Vivianne Freitas da Silva, Daniela Micheline dos Santos, Liliane da Rocha Bonatto, Victor Gustavo Balera Brito, Sandra Helena Penha de Oliveira, Marcelo Coelho Goiato

**Affiliations:** 1 Department of Dental Materials and Prosthodontics, Aracatuba Dental School, Sao Paulo State University (UNESP), Aracatuba, Sao Paulo, Brazil; 2 Department of Basic Sciences, Aracatuba Dental School, Sao Paulo State University (UNESP), Aracatuba, Sao Paulo, Brazil

**Keywords:** Eye, Materials testing, Methylmethacrylate

## Abstract

**Background:**

This study was undertaken to analyze if different preparation and exposure periods of eluates from ocular prosthesis acrylic resin influence the cytotoxicity for conjunctival cells.

**Methods:**

Twenty-four acrylic resin specimens were divided, according to the period of eluate exposure to Chang conjunctival cells (24 and 72 hours). Eluates were prepared in four different ways: 24, 48, and 72 hours of resin specimen immersion in medium and 24 hours of immersion in water, followed by 24 hours of immersion in medium. MTT assay was used to evaluate the cytotoxic effect. The production of IL-1β, IL-6, TNF-α, and chemokine macrophage inflammatory protein 1α was evaluated by ELISA, while the mRNA expression of type IV collagen (COL IV), transforming growth factor β (TGF-β), and matrix metalloproteinase 9 (MMP9) were evaluated by real-time RT-PCR technique. The statistical analysis was carried out using ANOVA with Bonferroni post-hoc test and the student’s *t*-test (*p* < 0.05).

**Results:**

Significant quantities of IL-6 (4.594 pg/mL) and mRNA expression of COL IV (1.58) were verified at 72 hours of eluate exposure to cells, as compared to 24 hours. After the 72-hour exposure of eluates to cells, lower cell proliferation (88.4%) and higher IL-6 quantities (12.374 pg/mL), as well as mRNA expression of COL IV (2.21), TGF-β (2.02), and MMP9 (5.75) were observed, which corresponded to 72 hours of a specimen immersed in medium.

**Conclusion:**

Longer periods of eluate preparation and exposure from the acrylic resin to cells are related to higher production of proinflammatory cytokines and extracellular matrix proteins.

## INTRODUCTION

The ocular prosthesis is an alternative treatment, aiming to improve facial aesthetics and self-esteem of patients with anophthalmia, as well as to restore and maintain the health of the remaining structures[1-3]. The N1 acrylic resin for artificial sclera and colorless acrylic resin is among the current materials applied for ocular prosthesis manufacturing[1,4]. This resin is widely used to fabricate the base of an ocular prosthesis due to its color similarity to the sclera and because of its durability, low cost, ease of cleaning, and mechanical retention in the anophthalmic cavity[4]. The polymerization of this material, which consists of a methyl methacrylate (MMA) polymer (powder) and an MMA monomer (liquid), allows the conversion of monomers into polymers with the optimization of physical properties[5,6].

Song *et al*.[7] have recommended that the prosthesis storage in water for 24 hours before installation in the patient could release the residual monomers and reduce mucosal irritation. However, Bettencourt *et al*.[8] have suggested that the release of monomers is continuous and can occur for years, even after the material polymerization.

For successful rehabilitation, prosthesis necessitates being non-toxic and biocompatible[9,10] and does not cause adverse effects locally or systemically on recipient[11]. *In vivo* cytotoxicity tests involve ethical issues for their implementation; therefore, *in vitro* tests are essential to ensure the safety of using ocular prosthesis material in humans. The cell culture method, for example, is a relatively simple test to perform, is reproducible, can be carefully controlled, and is adequate cost-benefit[9,12].

A colorimetric assay using a tetrazolium salt, MTT, can be performed for cytotoxicity analysis since it allows an indirect evaluation of cell proliferation through mitochondrial enzymatic activity of viable cells. In the present study, the possible cytotoxic effects of eluates from the synthetic materials were analyzed using MTT assay[13-16]. Primary cells or cell lines are necessary for evaluating the material cytotoxicity[9]. These cell lines are usually selected based on the highest similarity to the target organ. Furthermore, it is essential to examine the key mediators of inflammatory processes[17] and to evaluate the cytotoxicity of the acrylic resin, in order to ensure a safe clinical use of the prosthesis. Therefore, this study assessed if human conjunctival cells are influenced by different preparation and exposure periods of eluates from N1 ocular prosthesis acrylic resin, by evaluating the cell proliferation via MTT assay, the analysis of the production of proinflammatory cytokines through ELISA, and mRNA expression of extracellular matrix proteins through RT-PCR technique.

## MATERIALS AND METHODS

A total of 24 specimens of N1 acrylic resins (Artigos Odontológicos Clássico Ltda, Sao Paulo, Brazil) were manufactured, heat-polymerized in water bath and divided into two groups, according to the eluate exposure period to human conjunctival cell line (24 and 72 hours). A power analysis was performed to determine the number of specimens required for the study to provide sufficient power (>95%). Therefore, three specimens were selected per group.

Auto-polymerized resin disks, obtained from a metallic matrix containing 10 circular compartments of 10 mm in diameter and 3 mm in thickness, were used for specimen preparation[18]. These disks were positioned in flasks (Artigos Odontológicos Clássico Ltda) with the use of type IV dental stone (Durone, Dentsply Ind e Com Ltda, Rio de Janeiro, Brazil) and an extra hard laboratory silicon (Zhermack, Rovigo, Italy) for embedding the molds. After final setting of the materials, the flasks were opened, the disks were removed, and the molds were obtained[19,20].

The N1 acrylic resin specimens were proportioned and mixed according to the manufacturer’s instructions and placed in the mold-containing flasks. Subsequently, the counter flask was positioned and raised in a hydraulic bench press with a weight of 1.250 kgf for 2 minutes[21,22]. The polymerization was performed as per manufacturer’s instructions, initiated with bench polymerization after flask immersion in water at room temperature and maintained in mild heat for 30 minutes, followed by no heating for 30 minutes and then boiling for 1 hour. Later, the flasks were opened, and the excess specimen were removed with Maxi-Cut abrasive drill (Vicking, Sao Paulo, Brazil).

Eluates of substances leached from resin specimens were used for the analysis of their cytotoxic effect[12,23]. Three resin specimens from each group were placed, immediately after sample fabrication, into a sterile vial with 10 mL of Medium 199 (Gibco, New York, USA) supplemented with 10% FBS[12] and incubated at 37 ºC for periods of 24 (P1), 48 (P2), and 72 hours (P3). In addition, a period of immersion in distilled water for 24 hours followed by 24 hours of immersion in Medium 199 was evaluated (P4). There was a leaching of different substances from the matrix of the resin specimens for the culture medium during these periods, with the consequent formation of eluates that were incubated with cell cultures for conducting the cytotoxicity analysis[24]. After the incubation periods, the eluates were collected and filtered through 0.22-μm filters (Millex, Millipore, Darmstadt, Germany) for sterilization and prevention of the medium contamination[9,11].

For MTT assay, a human conjunctiva cell line (Wong Kilbourne derivative of Chang conjunctival cell line, clone 1-5c-4) was obtained from the American Type Culture Collection (CCL-20.2, Virginia, USA). Then the cells were expanded in flasks using Medium 199 supplemented with 10% FBS, 10 μg/mL of streptomycin, 10 μg/mL of penicillin, 10 μg/mL of gentamicin, and 250 μg/mL of fungizone. The culture was finally incubated with 5% CO_2_ in controlled humidity at 37 ºC[25-27].

Cell suspensions of 5 × 10^4^ cells/mL, predetermined by a pilot study, were prepared to perform the cytotoxicity tests, and 1 mL of this suspension was transferred into each well of a 24-well plate. After 24 hours of incubation with 5% CO_2_ and controlled humidity at 37 ºC, the medium was discarded, and 500 μL of eluates from different groups was added to each well. After 24 and 72 hours of eluate exposure to the cells, the culture medium was replaced by 500 μL of Medium 199 without FBS and with 0.5 mg/mL MTT. Subsequently, incubation with 5% CO_2_ at 37 ºC for 4 hours was performed[9,11,28]. The culture medium was removed, and the intracellular formazan was released by solubilization with 1 mL of isopropanol per well. The plates were shaken for 5 minutes before measuring the absorbance at 570 nm using a UV-visible spectrophotometer (SpectraMax 190, Molecular Devices, California, USA). The MTT assay was performed in triplicate[9,12,13,28].

The eluates obtained after four periods of specimen incubation (P1, P2, P3, and P4) were placed on the cell cultures, and the cell-free supernatants were collected after 24 and 72 hours. The purpose of the collection was to perform the dosage of pro-inflammatory cytokines, IL-β, IL-6, and TNF-α, as well as the chemokine macrophage inflammatory protein 1α (CCL3/MIP1α), by ELISA (DuoSet ELISA Development Systems, R&D System, Minnesota, USA)[25,29,30]. A total volume of 100 μL of cell-free supernatant was used for the quantitative analysis of the specimens performed in triplicate according to manufacturer’s recommendations[31].

The real-time reverse transcription-polymerase chain reaction (RT-PCR) was performed to analyze quantitatively the gene expression levels for type IV collagen (COL IV; COL4A3BP: Hs00178621_m1), matrix metalloproteinase 9 (MMP9; MMMP9: Hs00234579_m1), and transforming growth factor β (TGF-β; TGFB1: Hs0099133_m1)[29] in specimen incubation periods P2, P3, and P4. The analysis of the target genes by real-time RT-PCR at P1 was not performed since there was no significant reduction in cell proliferation or substantial increase in the levels of IL-6 and TNF-α in this period.

TRIzol reagent (Invitrogen Life Technologies, California, USA) was applied for total RNA extraction from the cells after 24 and 72 hours of eluate exposure to the cells, based on the instructions provided by the manufacturer. Spectrophotometry was employed to measure the RNA concentration. The first strand cDNAs were synthesized using 1 μg of total RNA and Superscript II RNase H^-^ reverse transcriptase (Invitrogen Life Technologies). Subsequently, the measurement of mRNA levels for COL IV, MMP9, and TGF-β, and their amplification by a StepOnePlus Real-Time PCR System (Applied Biosystems, Invitrogen Life Technologies) was conducted. The detection of mRNA for β-actin (ACTB: Hs03023880_g1) was used as the internal control. The reactions were performed using a volume of 20 μL, and each specimen was run in duplicate, following the thermal cycling conditions recommended by the manufacturer. The results were analyzed using the comparative threshold cycle (C_T_) method[10,25].

Data from the MTT, ELISA, and RT-PCR assays were submitted to two-way analysis of variance (ANOVA) for periods of preparation and exposure of eluates to cells, followed by Bonferroni post-tests with a 5% significance level. The student’s *t*-test with a 5% significance was used to evaluate differences between the eluate exposure periods to the conjunctival cell line (24 and 72 hours).

## RESULTS

**Fig. 1:**
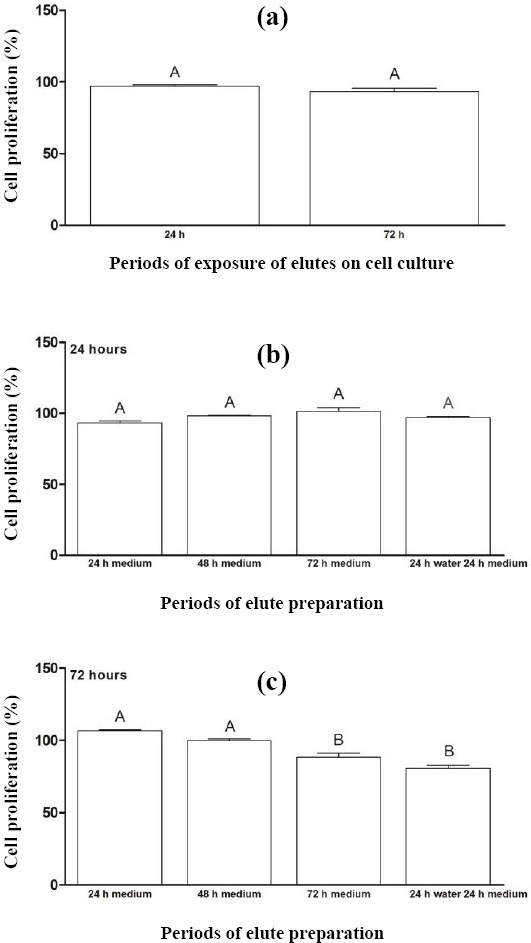
Percentage of cell proliferation (a) after the exposure of acrylic resin eluates to cell cultures for periods of 24 and 72 hours and for preparing eluates prior to their exposure to cell cultures for (b) 24 h and (c) 72 h. The results show mean ± standard error of cell proliferation percentage. Different capital letters for each chart indicate statistical difference (*p* < 0.05) between the periods analyzed.

Figure 1a shows the percentage of cell proliferation after the exposure of acrylic resin eluates to cell cultures for periods of 24 and 72 hours. There was no statistically significant difference between the periods (*p* = 0.146). Comparing different periods for preparing eluates before their exposure to cell cultures for 24 hours, no significant difference was observed (Fig. 1b). However, through Figure 1c, it can be seen that for the exposure period of 72 hours, lower percentages of cell proliferation were observed for eluate preparation periods P3 (88.4%) and P4 (80.5%), with statistically significant difference from other periods.

**Fig. 2:**
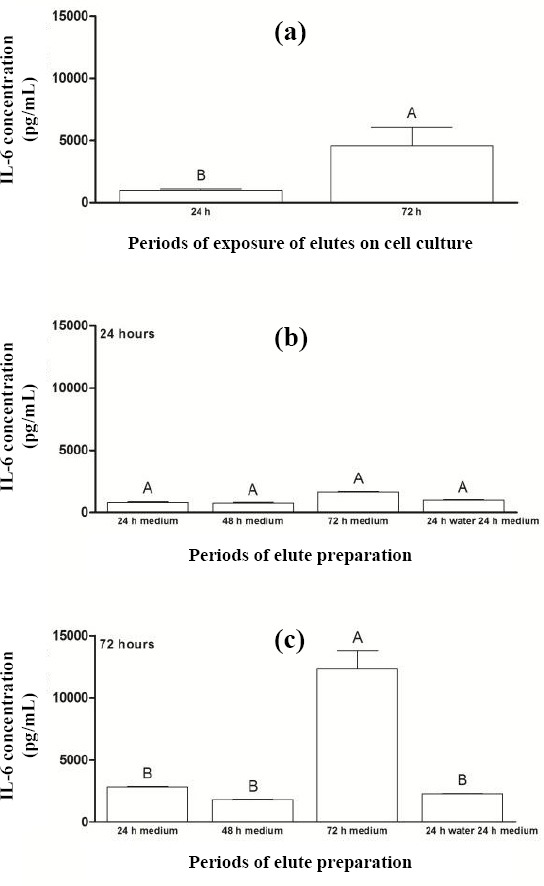
Concentration of IL-6 (a) after the exposure of acrylic resin eluates to cell cultures for periods of 24 and 72 hours and for preparing eluates prior to their exposure to cell cultures for (b) 24 h and (c) 72 h. The results show mean ± standard error of IL-6 concentration (pg/mL). Different capital letters for each chart indicate statistical difference (*p* < 0.05) between the groups.

Regarding the concentration of IL-1β and CCL3/MIP1α, detectable levels of these targets were not found in the present study. However, Figure 2a presents the concentration of IL-6 after the exposure of acrylic resin eluates to cell cultures for periods of 24 and 72 hours. Statistical difference was observed for the IL-6 concentration (*p* = 0.043) between the periods of 24 hours (385 pg/mL) and 72 hours (4.594 pg/mL). However, there was not any significant difference between different preparation periods of eluates before their exposure to cell cultures for 24 hours (Fig. 2b). On the other hand, when comparing the preparation periods before the exposure of eluates to cell cultures for 72 hours (Fig. 2c), we observed a higher concentration of IL-6 for eluate preparation period P3 (12.374 pg/mL), with statistically significant difference from other periods.

**Fig. 3:**
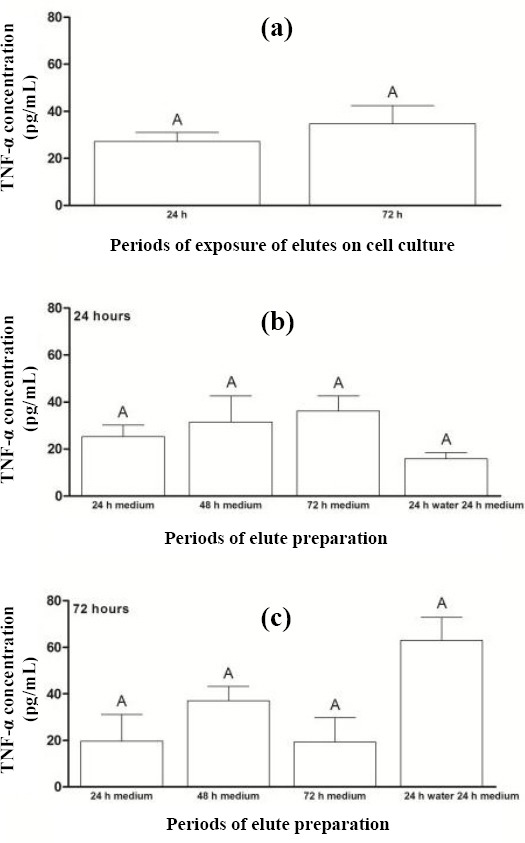
Concentration of TNF- α (a) after the exposure of acrylic resin eluates to cell cultures for periods of 24 and 72 hours and for preparing eluates prior to their exposure to cell cultures for (b) 24 h and (c) 72 h. The results show mean ± standard error of TNF-α concentration (pg/mL). Different capital letters for each chart indicate statistical difference (*p* < 0.05) between the groups.

**Fig. 4:**
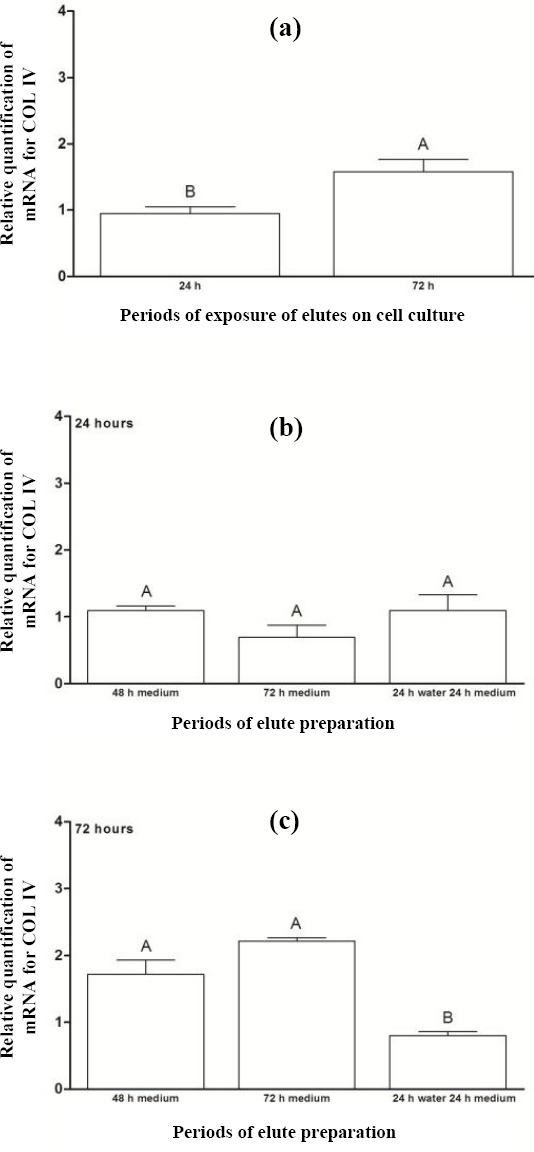
Relative quantification of mRNA for COL IV (a) after the exposure of acrylic resin eluates to cell cultures for periods of 24 and 72 hours and for preparing eluates prior to their exposure to cell cultures for (b) 24 h and (c) 72 h. The results show mean ± standard error of relative quantification of mRNA for COL IV. Different capital letters for each chart indicate statistical difference (*p* < 0.05) between the groups.

Figure 3 depicts the TNF-α concentration after the exposure of acrylic resin eluates to cell cultures for periods of 24 and 72 hours. There was no statistically significant difference in TNF-α concentration between the two mentioned periods (*p* = 0.394), regardless of the periods used for preparing eluates before their exposure to cell cultures. Figure 4a shows the relative quantification of mRNA for COL IV after the exposure of acrylic resin eluates to cell cultures for periods of 24 and 72 hours.

Statistical difference was observed (*p* = 0.01) between the periods of 24 hours (0.95) and 72 hours (1.58). For the different eluate preparation periods before their exposure to cell cultures for 24 hours (Fig. 4b), no statistical difference was noted between them. However, comparison of the preparation periods, before exposure of eluates to cell cultures, for 72 hours (Fig. 4c) demonstrated statistically higher COL IV expression for eluate preparation periods P2 (1.71) and P3 (2.21) relative to period P4.

**Fig. 5:**
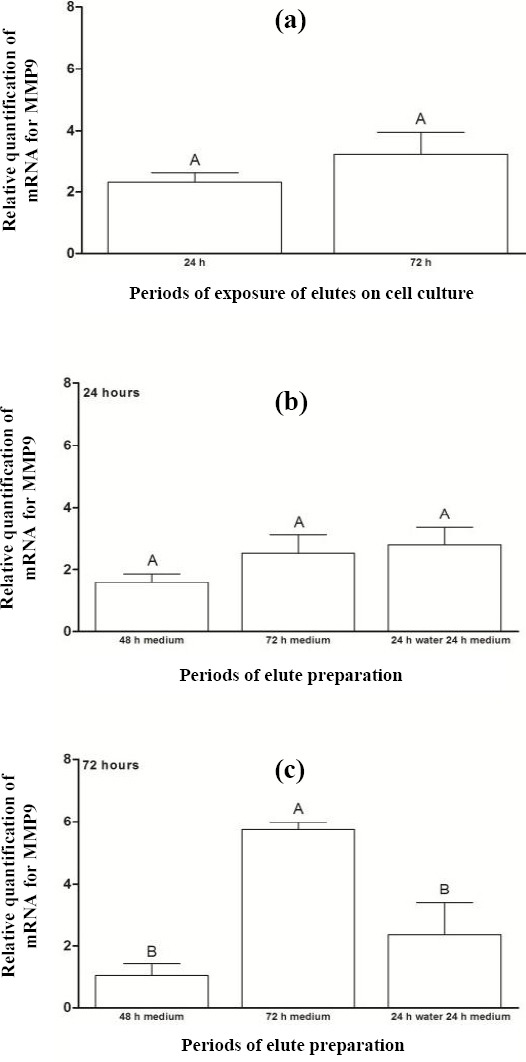
Relative quantification of mRNA for MMP9 (a) after the exposure of acrylic resin eluates to cell cultures for periods of 24 and 72 hours and for preparing eluates prior to their exposure to cell cultures for (b) 24 h and (c) 72 h. The results show mean ± standard error of relative quantification of mRNA for MMP9. Different capital letters for each chart indicate statistical difference (*p* < 0.05) between the groups.

The relative quantification of mRNA for MMP9 after 24 and 72 hours of exposure of acrylic resin eluates to cells is illustrated in Figure 5a. There was no statistically significant difference between the periods (*p* = 0.257), as well as among different periods for preparation of eluates before their exposure to cell cultures for 24 hours (Fig. 5b). On the other hand, comparison of the preparation periods before the exposure of eluates to cell cultures for 72 hours (Fig. 5c) verified a higher MMP9 expression for eluate preparation period P3 (5.75), with a statistically significant difference from other periods.

**Fig. 6:**
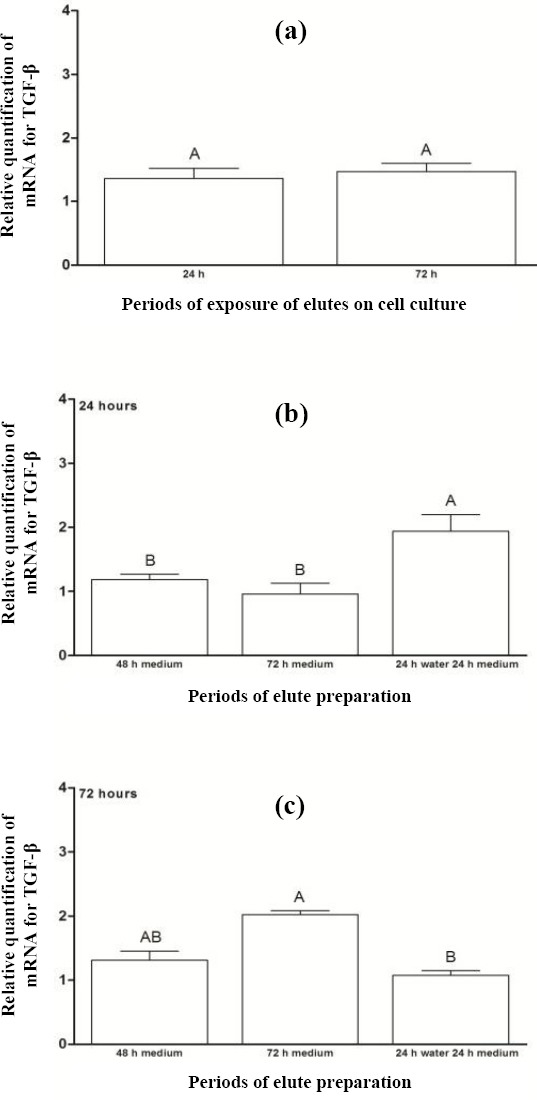
Relative quantification of mRNA for TGF-β (a) after the exposure of acrylic resin eluates to cell cultures for periods of 24 and 72 hours and for preparing eluates prior to their exposure to cell cultures for (b) 24 h and (c) 72 h. The results show mean ± standard error of relative quantification of mRNA for TGF-β. Different capital letters for each chart indicate statistical difference (*p* < 0.05) between the groups.

Figure 6a exhibits the relative quantification of mRNA for TGF-β after the periods of 24 and 72 hours of exposure of acrylic resin eluates to cells. No statistically significant difference was found between the mentioned periods (*p* = 0.606). Figure 6b shows comparison of preparation periods beforethe exposure of eluates to cell cultures for 24 hours. A higher TGF-β expression was observed for eluate preparation period P4 (1.94), with statistically significant difference from other periods. In addition, for the period of exposure of eluates to cell cultures for 72 hours (Fig. 6c), statistically higher TGF-β expression was found for eluate preparation period P3 (2.02), when compared to period P4.

## DISCUSSION

The null hypothesis tested in the present study, which states that different preparation and exposure periods of eluates from N1 ocular prosthesis color acrylic resin to the human conjunctival cell line do not produce cytotoxicity effects, was not accepted since the production of IL-6 (Fig. 2) and mRNA expression of COL IV (Fig. 4) was higher at 72 hours of eluate exposure to cells when compared to 24 hours. Regarding different preparation periods of eluates prior to their exposure to cell cultures, the longer the leaching of substances for the culture medium, the higher the production of proinflammatory cytokines and extracellular matrix proteins. Although statistical difference was observed only for the cited targets (IL-6 and COL IV) at 72 hours of exposure of acrylic resin eluates to cell cultures, higher concentrations of TNF-α and mRNA expression of MMP9 and TGF-β were found. The percentage of cell proliferation was lower in this period when compared to 24 hours. Cimpan *et al*.[32] have revealed that the higher concentration of soluble substances in the acrylic resin eluate and the longer exposure of this eluate to cell cultures, the greater the deleterious effects.

It is essential to emphasize that before performing the present study, we conducted a pilot study to determine the appropriate cell concentration (5 × 10^4^ cells/mL) for the cytotoxicity test, so that a monolayer of cells with confluent growth was observed at 72 hours of eluate exposure to cell cultures.

Regarding the different preparation periods of eluates before their exposure to cell cultures for 24 hours, there was no statistical difference between the eluate preparation periods (P1, P2, P3, and P4) for the percentage of cell proliferation or levels of IL-6 and TNF-α (Figs. 1b, 2b, and 3b). Similarly, no significant differences were observed between the eluate preparation periods P2, P3, and P4 for mRNA expression of COL IV and MMP9 (Figs. 4b and 5b). This result may be due to the shorter exposure period of acrylic resin eluates to cell cultures[32,33]. Concerning TGF-β, the highest expression was found for the period P4 (Fig. 6b).

According to Abbas *et al*.[17], TGF-β participates in the control of inflammatory response and tissue repair by stimulating collagen synthesis and promoting local angiogenesis. The metalloproteinase acts in degrading COL IV, as an extracellular matrix protein[34]. Therefore, the levels of TGF-β and MMP9 are required to be in balance during the tissue repair process. Based on Figures 5b and 6b, a balance was found in the expression of TGF-β and MMP9 for period P4.

Regarding the different preparation periods of eluates before their exposure to cell cultures for 72 hours, a lower percentage of cell proliferation was observed for periods P3 and P4 (Fig. 1c). Additionally, a higher concentration of IL-6 was found for period P3 when compared to other periods (Fig. 2c). This interleukin is one of the major proinflammatory cytokines, and along with TNF-α and IL-1, has the ability to increase the local concentration of tissue repair cells[17]. The reduction of cell proliferation and the increase in IL-6 concentration may be due to the release of different substances during the process, such as residual monomers. In fact, the aqueous medium penetrates the resin matrix and expands the gap between the polymer chains. As a result, residual monomers, which can be toxic to the cell, leach out of the material, causing an inflammatory process[24].

Two previous studies by Retamoso *et al*.[35] and Saravi *et al*.[36] have suggested that the maintenance of the acrylic resin in aqueous medium is essential for leaching of monomers before prosthesis installation in the patient, in order to reduce the deleterious effects of chemical irritation caused by MMA monomers. However, since the periods mentioned above showed similar results regarding the percentage of cell proliferation, it can be inferred that the immersion of ocular prosthesis acrylic resin in water for 24 hours before installation does not seem necessary for the reduction of cytotoxicity. However, the resin immersion in water is important to compensate the resin contraction[37].

According to the ISO 10993-5 standard, the degree of cytotoxic effect can be classified through *in vitro* methods for the cytotoxicity analysis as non-cytotoxic (cell proliferation higher than 75%), slightly cytotoxic (proliferation between 50 and 75%), moderately cytotoxic (proliferation between 25 and 50%), and highly cytotoxic (proliferation lower than 25%)[23]. Therefore, there was no cytotoxicity of acrylic resin for all eluate preparation periods analyzed (Fig. 1).

In the present study, there were no detectable concentrations of IL-1β and CCL3/MIP1α after the exposure of eluates from ocular prosthesis acrylic resin to Chang conjunctival cells. Therefore, we can conclude that this cell line could not be stimulated by the acrylic resin eluates tested for the production of those inflammatory mediators.

Regarding the relative quantification of mRNA for COL IV, MMP9, and TGF-β during different periods of the preparation of eluates before their exposure to cell cultures for 72 hours, larger amounts of these targets were observed in period P3 (Figs. 4c, 5c, and 6c). These data may result from the higher leaching of residual monomers for the culture medium[35,36,38]. Simon *et al*.[39] have evidenced that COL IV is produced by pulmonary alveolar epithelial cells, and its presence is necessary to compose the basement membrane, extracellular matrix. A balance in mRNA expression of COL IV, MMP9, and TGF-β is important, aiming to preserve the structure of the Chang conjunctival cell, which was used as the epithelial cell in this study. In the above-mentioned period, a similarincrease in gene expression levels of COL IV (Fig. 4c) and TGF-β (Fig. 6c) was observed; the latter protein stimulates the COL IV synthesis[17,34]. However, the mRNA expression of MMP9, which acts to degrade COL IV[17,34], increased approximately three times more than the expressions of COL IV and TGF-β (Fig. 5c), which may be deleterious since the excessive activation of MMP9 damages the cell morphology. According to Yang *et al*.[40], the overexpression of metalloproteinase can result in severe damage to tissue, and a balance between its activation and inhibition is necessary.

The current study analyzed the cytotoxicity and cell activation *in vitro*. Although the tests used in this study have limitations since the results do not fully reflect the cytotoxic properties of the material in their clinical condition, these assays are essential to determine the biocompatibility of ocular prosthesis in humans[6,12,36]. Further studies can be performed with the analysis of different inflammatory mediators and with the evaluation of longer release periods of water-soluble substances to obtain the extracts before their exposure to cell cultures.

A higher production of proinflammatory cytokines and extracellular matrix proteins results from a longer preparation and exposure periods of eluates from the N1 acrylic resin to the human conjunctival cells.
